# Biogenesis, conservation, and function of miRNA in liverworts

**DOI:** 10.1093/jxb/erac098

**Published:** 2022-03-11

**Authors:** Halina Pietrykowska, Izabela Sierocka, Andrzej Zielezinski, Alisha Alisha, Juan Carlo Carrasco-Sanchez, Artur Jarmolowski, Wojciech M Karlowski, Zofia Szweykowska-Kulinska

**Affiliations:** Department of Gene Expression, Institute of Molecular Biology and Biotechnology, Faculty of Biology, Adam Mickiewicz University, Uniwersytetu Poznanskiego 6, 61-614 Poznan, Poland; Department of Gene Expression, Institute of Molecular Biology and Biotechnology, Faculty of Biology, Adam Mickiewicz University, Uniwersytetu Poznanskiego 6, 61-614 Poznan, Poland; Department of Computational Biology, Institute of Molecular Biology and Biotechnology, Faculty of Biology, Adam Mickiewicz University, Uniwersytetu Poznanskiego 6, 61-614 Poznan, Poland; Department of Gene Expression, Institute of Molecular Biology and Biotechnology, Faculty of Biology, Adam Mickiewicz University, Uniwersytetu Poznanskiego 6, 61-614 Poznan, Poland; Department of Computational Biology, Institute of Molecular Biology and Biotechnology, Faculty of Biology, Adam Mickiewicz University, Uniwersytetu Poznanskiego 6, 61-614 Poznan, Poland; Department of Gene Expression, Institute of Molecular Biology and Biotechnology, Faculty of Biology, Adam Mickiewicz University, Uniwersytetu Poznanskiego 6, 61-614 Poznan, Poland; Department of Computational Biology, Institute of Molecular Biology and Biotechnology, Faculty of Biology, Adam Mickiewicz University, Uniwersytetu Poznanskiego 6, 61-614 Poznan, Poland; Department of Gene Expression, Institute of Molecular Biology and Biotechnology, Faculty of Biology, Adam Mickiewicz University, Uniwersytetu Poznanskiego 6, 61-614 Poznan, Poland; University of Parma, Italy

**Keywords:** Conserved and non-conserved miRNAs in plants, liverworts, miRNA biogenesis, *MIR* genes, microprocessor, proteins involved in miRNA biogenesis

## Abstract

MicroRNAs (miRNAs) are small non-coding endogenous RNA molecules, 18–24 nucleotides long, that control multiple gene regulatory pathways via post-transcriptional gene silencing in eukaryotes. To develop a comprehensive picture of the evolutionary history of miRNA biogenesis and action in land plants, studies on bryophyte representatives are needed. Here, we review current understanding of liverwort *MIR* gene structure, miRNA biogenesis, and function, focusing on the simple thalloid *Pellia endiviifolia* and the complex thalloid *Marchantia polymorpha.* We review what is known about conserved and non-conserved miRNAs, their targets, and the functional implications of miRNA action in *M. polymorpha* and *P. endiviifolia*. We note that most *M. polymorpha* miRNAs are encoded within protein-coding genes and provide data for 23 *MIR* gene structures recognized as independent transcriptional units. We identify *M. polymorpha* genes involved in miRNA biogenesis that are homologous to those identified in higher plants, including those encoding core microprocessor components and other auxiliary and regulatory proteins that influence the stability, folding, and processing of pri-miRNAs. We analyzed miRNA biogenesis proteins and found similar domain architecture in most cases. Our data support the hypothesis that almost all miRNA biogenesis factors in higher plants are also present in liverworts, suggesting that they emerged early during land plant evolution.

## Teaser

This review highlights recent findings on the liverwort miRNA repertoire, including *Marchantia polymorpha*miRNA gene organization, biogenesis, and functions, and microprocessor, auxiliary, and regulatory proteins involved in miRNA biogenesis.

## Introduction

Since the discovery of microRNAs (miRNAs) in the early 1990s, these small non-coding RNAs have been found in almost all eukaryotic lineages (Lee *et al.*, 1993; Carrington and Ambros, 2003; Moran *et al.*, 2017; Y. Zhang *et al.*, 2018). miRNAs are involved in various biological processes, including cell division, expansion, and differentiation, as well as in the specification of organ identity, developmental phase transitions, responses to environmental stresses, and many others (Li and Zhang, 2016; Liu *et al.*, 2018). Our knowledge of miRNA function in plants has been derived mainly from studies of model plants with sequenced genomes, most of which are angiosperms; thus, our knowledge relies on only one of the major land plant lineages.

**Fig. 1.**
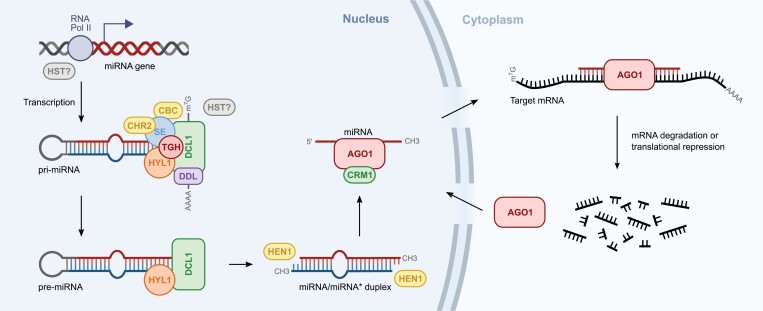
miRNA biogenesis in plants. RNA polymerase II (RNA Pol II) transcribes miRNA genes. Primary transcripts (pri-miRNAs) contain a cap at their 5ʹ end and are polyadenylated at their 3ʹ end. The activity of the DCL1 endonuclease first cuts off the imperfectly folded stem-and-loop structure of pri-miRNAs, resulting in a shorter stem-loop hairpin (pre-miRNA). This reaction entails the concerted action and physical interactions of SE, HYL1, DCL1, and CBC. Throughout the process, several auxiliary proteins participate in the interactions with the microprocessor core, among others TGH, DDL, and CHR2. The resulting pre-miRNAs are further excised by DCL1 to mature miRNA/miRNA* duplexes. Next, the 3ʹ-ends of miRNA/miRNA* duplexes are methylated by HEN1. The guide miRNA strand is then integrated into AGO1 protein with the aid of CRM1 protein and exported to the cytoplasm, where it regulates the cognate mRNA level.

miRNAs are endogenous molecules 18–24 nucleotides in length that repress gene expression via mRNA cleavage or translational inhibition of targets. The miRNA-induced silencing complex (miRISC) is guided to a target mRNA by the complementary binding of a miRNA to its target mRNA (Bartel, 2009; Rogers and Chen, 2013; Yu *et al.*, 2017). In higher plants, most miRNA genes (*MIR*s) are independent transcription units, with the rest being located within the introns of protein-coding or non-coding genes (Stepien *et al.*, 2017). All plant *MIR* genes studied to date are transcribed by RNA polymerase II (RNA Pol II), which produces primary transcripts (pri-miRNAs) that are 5ʹ capped and 3ʹ polyadenylated. The characteristic feature of pri-miRNAs is that they can fold back into hairpin-like structures that house miRNA/miRNA* (miRNA strand/passenger strand) duplexes within the stem of the hairpin (Xie *et al.*, 2005; Wang *et al.*, 2019; Li and Yu, 2021). The process of maturation of the plant pri-miRNA to the final miRNA molecule takes place entirely in the cell nucleus, where it is directed by a multiprotein microprocessor in a two-step process (Fig. 1). First, the stem-loop structure of the pri-miRNA causes an initial cleavage event that releases the precursor miRNA (pre-miRNA) hairpin. The pre-miRNAs are then processed further into miRNA/miRNA* duplexes during the second step of miRNA biogenesis (Bologna *et al.*, 2009; Cuperus *et al.*, 2010; Mateos *et al.*, 2010; Song *et al.*, 2010; Werner *et al.*, 2010; Wang *et al.*, 2019; Li and Yu, 2021). Here, both endonucleolytic cleavage events are catalyzed by a RNase III type enzyme, Dicer-Like 1 (DCL1), which recognizes the stem-loop structures in both pri-miRNAs and pre-miRNAs (Park *et al.*, 2002; Sun *et al.*, 2018; Wei *et al.*, 2021). For the efficient and correct processing of plant pri-miRNAs by DCL1, the assistance of microprocessor core proteins, including the double-stranded RNA-binding protein Hyponastic Leaves 1 (HYL1) and the zinc finger protein SERRATE (SE), is needed (Han *et al.*, 2004; Lobbes *et al.*, 2006; Yang *et al.*, 2006; Dong *et al.*, 2008; Achkar *et al.*, 2018; Wang *et al.*, 2019; Xie *et al.*, 2021). The final products of plant microprocessor action, miRNA/miRNA* duplexes, are further methylated at the 3ʹ ends by Hua Enhancer 1 methylase (HEN1), which provides protection against degradation after export from the nucleus to the cytoplasm (Yang *et al.*, 2006; Plotnikova *et al.*, 2013; Baranauske *et al.*, 2015). 

In the last step of miRNA biogenesis, the miRNA/miRNA* duplex is incorporated into an Argonaute 1 (AGO1) miRISC. The miRNA* is then removed, leaving only the miRNA, which is stabilized within the miRISC complex (Vaucheret *et al.*, 2004; Baumberger and Baulcombe, 2005; Yu *et al.*, 2017; Tomassi *et al.*, 2020). For a long time, it was hypothesized that the methylated miRNA/miRNA* duplexes are exported to the cytoplasm by the exportin Hasty (HST) (Park *et al.*, 2005). Interestingly, recent studies show that the miRISC complex is assembled in the nucleus and then exported to the cytosol in a Chromosomal Maintenance 1 (CRM1; also named EXPORTIN1)/nuclear-export-dependent manner (Fig. 1) (Bologna *et al.*, 2018; B. Zhang *et al.*, 2020). In support of this model, a recent study by Cambiagno *et al.* (2021) reported that HST is associated with the formation of the miRNA biogenesis complex for a specific subset of *MIR* genes. This permits the promotion of pri-miRNA transcription and processing rather than the direct export of processed miRNAs from the nucleus. In addition, HST has also been shown to be autonomously required for facilitating both cell-to-cell and phloem-based long-distance movement of fully processed miRNAs (Brioudes *et al.*, 2021). Thus, based on the latest data, AGO1 is the main protein responsible for miRNA export from the nucleus to the cytoplasm.

During the past decade, the development of high-throughput sequencing has enabled the identification of numerous miRNAs sourced from various non-model plant species, from red and green algae to seed plants (Molnár *et al.*, 2007; Amborella Genome Project, 2013; Alaba *et al.*, 2015; Xia *et al.*, 2015; Valli *et al.*, 2016; Cock *et al.*, 2017; Noronha Fernandes-Brum *et al.*, 2017; Ortiz *et al.*, 2019). Interestingly, among the data present in the literature and in available land plant sequence databases, miRNA repertoire characterization has been performed for only four bryophyte species, comprising one hornwort, two liverworts, and one moss (Khraiwesh *et al.*, 2010; Arazi, 2012; Alaba *et al.*, 2015; Lin *et al.*, 2016; Tsuzuki *et al.*, 2016; You *et al.*, 2017; J. Zhang *et al.*, 2020). This contrasts with the data available for 75 angiosperm species, as registered in miRBase version 22 (Kozomara *et al.*, 2019).

According to the available data, the main molecular framework responsible for the biogenesis, degradation, and mode of action of miRNAs was likely already established in the common ancestor of all land plants; this is because major components of the miRNA pathway have been identified in all studied bryophytes (Arif *et al.*, 2012, 2022; Lin *et al.*, 2016; Tsuzuki *et al.*, 2016; You *et al.*, 2017). However, the almost complete lack of mechanistic studies on miRNA biogenesis in bryophytes impedes our understanding of miRNA production in these plants.

While the precise phylogenetic relationships among the bryophytes remain unresolved, the critical position of liverworts—one of the earliest land plant lineages—is unequivocal (Puttick *et al.*, 2018). Therefore, liverworts represent a key group that comparative genomics can use to resolve fundamental questions in plant evolution. In this review, we summarize findings from studies of miRNAs and genes related to miRNA biogenesis from representatives of two liverwort lineages: *Pellia endiviifolia*, with simple thallus morphology (simple thalloid liverwort), and *Marchantia polymorpha*, which shows functional specialization within specific thallus sections/parts (complex thalloid liverwort). Furthermore, we present an analysis of *M. polymorpha MIR* gene structures based on the available genomic and transcriptomic data. We also provide new insights regarding the conservation of miRNA biogenesis machinery by focusing on both the core processing machinery components that have been described in *M. polymorpha* and by identifying the auxiliary and regulatory proteins influencing the folding, stability, and/or processing of pri-miRNAs.

## Identification of conserved land plant miRNAs present in liverworts

The first report describing the liverwort miRNA repertoire came from *P. endiviifolia*, in which combined analyses of the transcriptome, small RNAs (sRNAs), and degradome provided experimental evidence for target mRNA turnover by identified conserved and novel miRNAs. In total, 486 miRNAs belonging to 345 families (311 conserved and 34 novel families) were identified; these included numerous vascular-plant-specific miRNAs, of which 10 conserved miRNA families were found to be common to all land plants (miR156, miR160, miR166, miR171, miR319/159, miR390, miR395, miR396, miR408, and miR535) (Alaba *et al.*, 2015).

Small RNA profiling was also used to identify miRNAs and their precursors by comparing the sRNA sequencing data with genomic DNA and transcriptome profiles in *M. polymorpha*. Based on the obtained data, 265 miRNAs were identified and were mapped to 124 genomic loci (Tsuzuki *et al.*, 2016; Bowman *et al.*, 2017). In *M. polymorpha*, only seven miRNAs (miR160, miR166, miR171, miR319/miR159, miR390, miR408, and miR529) are conserved with those of other land plants (Tsuzuki *et al.*, 2016; Bowman *et al.*, 2017).

**Fig. 2.**
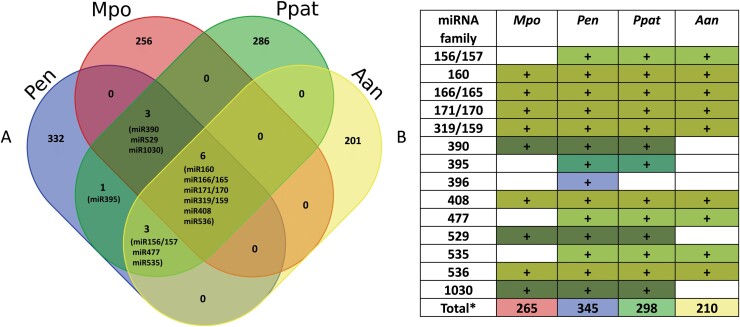
miRNA families shared within Bryophyta. (A) Venn diagram showing the number of total (conserved and species-specific) miRNA families identified in *P. endiviifolia* (Pen), *M. polymorpha* (Mpo), *P. patens* (Ppat), and *A. angustus* (Aan), according to Alaba *et al.* (2015), Tsuzuki *et al.* (2016), Bowman *et al.* (2017), and J. Zhang *et al.* (2020). The information about the total number of *P. patens* miRNAs was taken from miRBase v22 (Kozomara *et al.*, 2019); (B) Conserved miRNA families within Bryophyta. * Number of total (conserved and species-specific) miRNA families. The colors used in the table correspond to the colors in (A).

As liverworts belong to the bryophytes, we analyzed conserved miRNAs within this group (Fig. 2). Only six conserved miRNA families (miR319/159, miR160, miR166/165, miR171/170, miR408, and miR536) are shared by the hornwort *Anthoceros angustus*, the liverworts *P. endiviifolia* and *M. polymorpha*, and the moss *Physcomitrium patens*. Moreover, an additional three conserved miRNA families are shared between *P. endiviifolia*, *Physcomitrium*, and *Anthoceros* (miR156/157, miR477, and miR535), and three miRNA families (miR390, miR529, and miR1030) are shared by *M. polymorpha*, *P. endiviifolia*, and *Physcomitrium* (Alaba *et al.*, 2015; Tsuzuki *et al.*, 2016; Bowman *et al.*, 2017; Kozomara *et al.*, 2019; Li *et al.*, 2020; J. Zhang *et al.*, 2020). Our analysis suggests that a specific set of six conserved miRNAs common to all bryophytes and higher plants studied so far probably arose before the emergence of a common ancestor of all land plants (Deng *et al.*, 2017; Yang *et al.*, 2019). Six miRNA families that are highly conserved between these two groups also target mostly conserved mRNAs (miR536 seems to target different mRNAs in bryophytes and higher plants). This implies that they play similar and important roles in functional regulatory networks that coordinate plant development in all land plants (Tsuzuki *et al.*, 2016; Xia *et al.*, 2016; Lin and Bowman, 2018; Guo *et al.*, 2020).

## Liverwort-specific miRNAs shared by *M. polymorpha* and *P. endiviifolia*

**Fig. 3.**
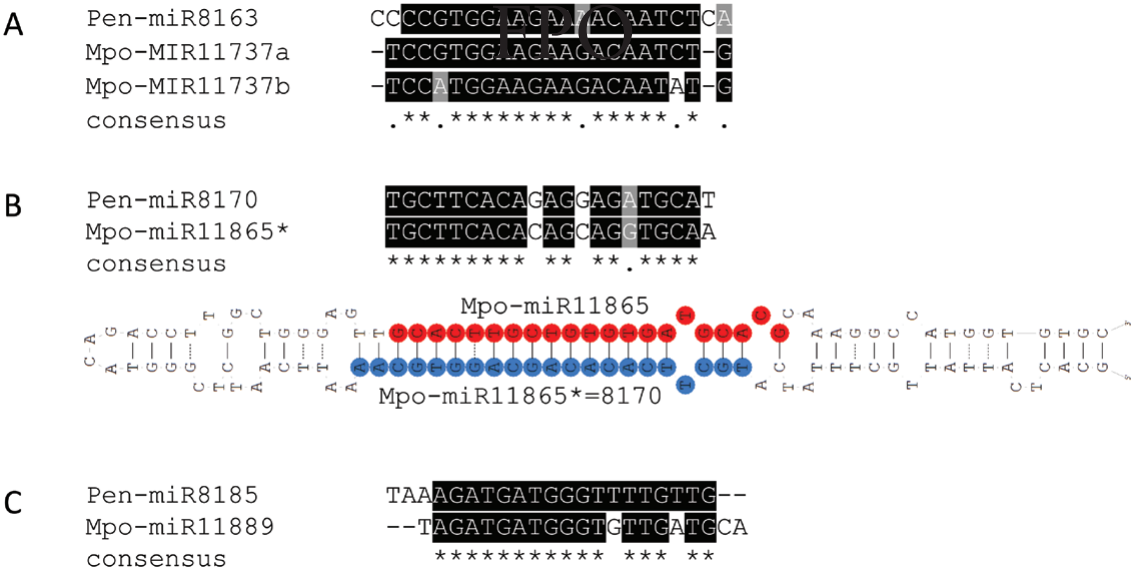
Sequence alignments of liverwort-specific miRNAs. (A) Sequence alignments of *P. endiviifolia* Pen-miR8163 and *M. polymorpha* Mpo-miR11737a and Mpo-miR11737b; (B) Upper panel - Pen-miR8170 and putative Mpo-miR11865* corresponding to Pen-miR8170, lower panel - structure of *M. polymorpha* pre-Mpo-miR11865 with designated Mpo-miR11865 and putative MpomiR8170; (C) Sequence alignments of Pen-miR8185 and Mpo-miR11889. Pen – *Pellia endiviifolia*, Mpo – *Marchantia polymorpha*.

An earlier comparison of the set of miRNAs found in the two liverwort species *M. polymorpha* and *P. endiviifolia* revealed no overlap in non-conserved miRNAs, with one exception. This was the single *P. endiviifolia*-specific Pen-miR8163, which shares a similar sequence with the *M. polymorpha*-specific Mpo-miR11737a. miR8163 in *P. endiviifolia* seems to represent a single-member family, whereas in *M. polymorpha* the Mpo-miR11737 family consists of two members, Mpo-miR11737a and Mpo-miR11737b (Fig. 3A) (Tsuzuki *et al.*, 2016). Next-generation sequencing results suggest that Mpo-miR11737a is more abundant than Mpo-miR11737b. Furthermore, these data also show that Mpo-miR11737a is up-regulated in male vegetative thalli (Tsuzuki *et al.*, 2016). However, the detailed expression profile of its *P. endiviifolia* ortholog, Pen-miR8163, is not known (Zielezinski *et al.*, 2015).

Analysis of the *M. polymorpha* genome (MarpolBase database) revealed the presence of a short RNA sequence similar to Pen-miR8170. Interestingly, this sRNA is encoded within the precursor of the *M. polymorpha*-specific Mpo-miR11865, and thus represents the miRNA* of Mpo-miR11865 (Fig. 3B) (Tsuzuki *et al.*, 2016; Bowman *et al.*, 2017). Moreover, it has been found that the expression of Mpo-miR11865 is relatively low in *M. polymorpha*, and its miRNA* was not annotated as the miRNA* of Mpo-miR11865. Thus, it cannot be excluded that the miRNA* of Mpo-miR11865 also acts as a miRNA, as it does in *P. endiviifolia*.

We also identified Mpo-miR11889, which is a homolog of Pen-miR8185 (Fig. 3C). Interestingly, Mpo-miR11889 is strongly up-regulated in *M. polymorpha* antheridiophores, similarly to Pen-miR8185, which is up-regulated in male generative thalli (Zielezinski *et al.*, 2015; Tsuzuki *et al.*, 2016). Careful inspection of microtranscriptomic data for *M. polymorpha* and *P. endiviifolia* thus has revealed the presence of three miRNAs common to the genera *Marchantia* and *Pellia*. Given that *Pellia* and *Marchantia* shared a last common ancestor ~350 million years ago (i.e. in the Paleozoic era), the presence of miRNAs common to both lineages suggests that they play important roles in liverwort physiology. Moreover, finding only three miRNAs common to *P. endiviifolia* and *M. polymorpha* supports a model of frequent gain and loss of *MIR* genes during evolution, as has been previously suggested (Fahlgren *et al.*, 2007; Axtell *et al.*, 2007).

## The function of liverwort miRNAs

In recent years, significant progress has been made in characterizing the biological roles of key miRNAs in various plant species. To fully understand the mode of action of miRNAs, it is imperative to define miRNA targets. In the first part of this section, we sum up what is known about miRNA–target modules when comparing liverworts with other land plants, and in the second part we focus on the effects of miRNAs on the development and physiology of *M. polymorpha*.

### Evolutionary conservation of miRNAs and their targets in liverworts

Analyses of miRNAs and their targets in *P. endiviifolia* and *M. polymorpha* revealed conserved as well as fluid miRNA–target relationships. In both liverwort species three conserved miRNAs, miR160, miR166, and miR408, have been demonstrated to target orthologous genes from the Auxin Response Factor (ARF), Class III Homeodomain-Leucine Zipper (HD-ZIP III), and plantacyanin families, respectively (Floyd and Bowman, 2004; Alaba *et al.*, 2015; Lin *et al.*, 2016). Similarly to angiosperms, heavy metal ATP synthase protein 8 (ATPase 8) and ppa004802m targeted by miR408 were also found as conserved targets in *P. endiviifolia* (Alaba *et al.*, 2015). In *M. polymorpha*, the conserved miRNAs miR171, miR319, and miR529 were shown to guide mRNA cleavage of the same target gene families as found in other land plants; these gene families included Gibberellic Repressor Scarecrow (GRAS), Transcriptional activator Myb (MYB), and Squamosa Promoter Binding Protein-Like (SPL) transcription factors, respectively (Lin *et al.*, 2016; Tsuzuki *et al.*, 2016; Flores-Sandoval *et al.*, 2018a; Lin and Bowman, 2018). The identified examples of conserved miRNA–target nodes in liverworts support previous findings indicating that the evolutionarily conserved miRNA–target pairs are crucial for the regulation of ancestral transcription factors or physiological enzymes involved in basic plant development or tolerance to stresses (You *et al.*, 2017; Axtell and Meyers, 2018).

A large number of target mRNAs identified in liverworts appear to be unique to each of the species studied, a phenomenon also observed in other land plants. Still, many of these non-conserved miRNA–target dependencies may play important roles for proper *P. endiviifolia* or *M. polymorpha* growth and development, as newly identified targets mostly encode nucleic acid-binding proteins and proteins with various catalytic activities (Alaba *et al.*, 2015; Lin *et al.*, 2016; Tsuzuki *et al.*, 2016). Several *M. polymorpha* transcription factors whose orthologs in higher plants regulate developmental processes (including MADS-box, homeodomain, and zinc finger transcription factors) have been found to be under the control of *M. polymorpha*-specific miRNAs (Mpo-miR11681, -miR11687, -miR11693, and -miR11677). Furthermore, *M. polymorpha* orthologs of angiosperm genes known to be involved in the development of the meristem (*Barely Any Meristem 2*; *AtBAM2*) and the epidermal layer of the seed (*Defective Kernel 1*; *AtDEK1*) were also identified as targets of the *M. polymorpha-*specific miRNAs Mpo-miR11687 and Mpo-miR11677, respectively (Lin *et al.*, 2016). However, the *Arabidopsis thaliana* transcripts *AtBAM2* and *AtDEK1* are known to not be under miRNA control. Thus, the role of *M. polymorpha* BAM and DEK1 ortholog proteins is an interesting question that has yet to be answered. Efforts to understand the *M. polymorpha*-specific miRNA–target interactions will help to elucidate how these species-specific miRNAs affect specific growth processes in *M. polymorpha*. Moreover, these data indicate that during the evolution of liverworts, miRNA-mediated regulation of gene expression was implemented to control diverse developmental processes.

Interestingly, degradome data have shown that two novel miRNAs, Mpo-miR11707.1 and Mpo-miR11707.2, are produced from a single precursor and target the Mp*AGO1* transcript (Lin *et al.*, 2016). Similarly, in the moss *P. patens* miR904 targets three *PpAGO1* homologs (*PpAGO1a–c*), whereas in *A. thaliana* the single *AtAGO1* mRNA is targeted by miR168 (Floyd and Bowman, 2004; Lin *et al.*, 2016; Tsuzuki *et al.*, 2016; Flores-Sandoval *et al.*, 2018a; Lin and Bowman, 2018). Taken together, these data suggest that miRNA-mediated feedback regulation of the miRNA biogenesis pathway evolved independently in the bryophyte and angiosperm lineages, with different miRNAs regulating the same target, namely AGO1 mRNA. These data underline how important it is to control the miRNA level in plants and that AGO1 feedback regulation plays a crucial role in this process.

The available literature shows that only a small number of the evolutionarily conserved miRNA–target modules have been identified in bryophytes and higher plants. Nonetheless, the majority of liverwort-identified miRNAs appear to target transcription factor mRNAs that are involved in various developmental processes, signaling, and stress response pathways, just as they do in angiosperms. Encouragingly, growing evidence shows that non-conserved miRNA–target nodes are also regulators of gene expression and function as crucial determinants of plant growth and development.

### Physiological relevance of the miRNA action in *M. polymorpha* development

To date, only a few functional studies of miRNA action have been published for liverworts. One of the first studies was by Tsuzuki *et al.* (2016), who demonstrated the effects of ectopic miRNA expression on *M. polymorpha* development. Plants overexpressing miR166a and miR319b exhibited clear morphological phenotypic changes, namely curling of the thalli to the ventral or dorsal side, respectively. In addition, the ectopic expression of either miRNA triggered the absence of gemma cups, although gemmae were produced in miR319b- overexpressing plants. While miR166a overexpression reduced the transcript levels of the predicted target gene, Mp*C3HDZ*, this Mp*MYB-like* transcript was not affected by miR319b overexpression. In addition, rapid amplification of cDNA ends (RACE) experiments have shown that Mp*MYB-like* mRNA is cleaved at the predicted miR319b cleavage site in *M. polymorpha* (Tsuzuki *et al.*, 2016). The authors proposed two possible explanations: (i) miR319b repression of Mp*MYB33-like* gene transcript may occur mainly at the translational level, like *APETALA2* repression by miR172 in Arabidopsis (Aukerman and Sakai, 2003; Tsuzuki *et al.*, 2016); or (ii) another, as yet uncharacterized, miR319b target is implicated in this regulatory mode of action during *M. polymorpha* development. Overall, this study revealed that miR166a and miR319b play important roles in the proper horizontal expansion of thalli and in the differentiation of gemma cups in *M. polymorpha* (Tsuzuki *et al.*, 2016).

In another study, it was shown that Mp*ARF3*, encoding class C Auxin Response Factor, is the primary gene regulated by miR160. Both loss- and gain-of-function Mp*ARF3* alleles resulted in pleiotropic defects manifested throughout the gametophytic stages of the life cycle. This indicated that Mp*ARF3* is a negative regulator of differentiation and promoter of cell proliferation. Mp*arf3* knockout mutants exhibited smaller cell size, more differentiated air pores, the production of a greater number of pegged rhizoids, and more gametophores per thallus area, with ectopic antheridia and fused archegoniophores. Conversely, Mp*mir160* knockout mutants seemed to lack proper differentiation of air pores, gemma cups, or gametophores under inductive conditions and produced more branched and convoluted thalli. These observations indicate that the MpARF3 protein and miR160 play roles as specific modulators of the cell differentiation rate and totipotency in *M. polymorpha* (Flores-Sandoval *et al.*, 2018a). Next, a differential gene expression analysis of Mp*arf3*/Mp*mir160* mutant plant transcriptomes by Flores-Sandoval *et al.* (2018b) identified a set of genes that were genuinely dependent on the Mp*ARF3*/Mp*MIR160* regulatory module. Of these, two genes belonging to the *SPL* transcription factor family were identified as repressed by MpARF3. At the same time, two miRNA precursors in which Mpo-miR11671 and Mpo-miR529c are embedded and which target Mp*SPL1* and Mp*SPL2* gene transcripts, respectively, were found to be activated by MpARF3. In angiosperms, it is known that SPL proteins are involved in controlling the transition from vegetative to reproductive life stages. By combining their results with data from the literature, it was pointed out that MpARF3 may antagonize the reproductive transition during the life cycle of *M. polymorpha*. This hypothesis seems plausible, given that the specific expression pattern of both Mp*SPL* genes has been observed along with an explicit expression peak in gametangiophores and a simultaneous down-regulation of both miRNA precursors at this developmental stage (Flores-Sandoval *et al.*, 2018b). Indeed, Tsuzuki *et al.* (2019) showed that miRNA529c regulates the reproductive transition in *M. polymorpha* by repressing Mp*SPL2* gene expression during vegetative growth, thereby impeding the appearance of reproductive branches and reproductive organs. Analysis of Mp*spl2* knockout mutant lines suggests that MpSPL2 may also play a role in the promotion of the reproductive transition and is needed for the proper development of reproductive branches. However, the Mp*SPL2* gene is not essential for gamete differentiation or gamete function, as crosses between male and female Mp*spl2* plants resulted in viable spore production (Tsuzuki *et al.*, 2019). Thus, the miR156/529–SPL module seems to control the vegetative-to-reproductive phase change in bryophyte development, as it does in angiosperms. However, further investigation of the Mp*SPL1*/Mpo-*MIR11671* regulatory module is necessary.

Bryophyte rhizoids have been found to be morphologically similar to vascular plant root hairs (Honkanen *et al.*, 2018). Basic helix-loop-helix (bHLH) transcription factors encoded by *Root Hair Defective Six-like* (*RSL*) class I genes have been shown to be positive regulators of both root hair development in vascular plants and rhizoid development in non-vascular plants (Menand *et al.*, 2007; Proust *et al.*, 2016; Kim *et al.*, 2017). Interestingly, Honkanen *et al.* (2018) identified a negative regulator of the *M. polymorpha* Mp*RSL1* gene that encodes a novel miRNA, Mp*Few Rhizoids1* (Mp*FRH1*) (previously described as Mpo-miR11861 by Tsuzuki *et al.*, 2016). A comparison of Mp*FRH1* gain-of-function and loss-of-function mutant plant phenotypes revealed decreases and increases, respectively, in the mRNA levels of the predicted target gene, Mp*RSL1*. In addition, RACE experiments also showed that the Mp*RSL1* transcript is cleaved within the Mp*FRH1* miRNA recognition site. Although *RSL* class I genes in Arabidopsis are negatively regulated by the homeodomain protein Glabra2 (AtGL2), there is no known miRNA involved in the regulation of *AtRSL* in Arabidopsis (Proust *et al.*, 2016; Honkanen *et al.*, 2018). Therefore, the discovery of a new species-specific miRNA in *M. polymorpha* revealed a different and independent mechanism of repression of this *RSL* class I gene despite the fact that its function is conserved compared with other land plants (Honkanen *et al.*, 2018).

Taken together, the studies described here revealed the first insights into the regulatory role played by miRNAs in liverworts. However, despite increasing interest in studies of the microtranscriptomes of bryophytes in the recent past, new research is required to elucidate both the compositional and the functional implications of the miRNA–target modules in bryophytes.

## *M. polymorpha* miRNAs are mainly encoded in protein-coding genes

Previous research found that *M. polymorpha* miRNAs are encoded either between or within protein-coding genes (Lin *et al.*, 2016; Bowman *et al.*, 2017). We therefore examined various transcriptomic datasets covering both miRNA precursors and mature miRNAs to analyse their coding position. Sequence annotations for both the precursor and mature miRNAs of *M. polymorpha* were retrieved from the GFF annotation file of the MpTak v6.1 reference genome assembly (Montgomery *et al.*, 2020). These annotations covered 256 pre-miRNAs and 319 miRNAs. We used BEDTools to retrieve a list of 107 pre-miRNAs whose nucleotide sequences did not overlap with other annotated genes and thus may represent independent transcription units (Quinlan and Hall, 2010). The other 149 pre-miRNAs overlapped with protein-coding genes (Table S1 at Zenodo; Pietrykowska *et al.*, 2022). From this sample, 95 pre-miRNAs were found to reside in genes encoding proteins that are orthologs of known proteins or that possess known protein motifs. These 95 pre-miRNAs were found in 5ʹ or 3ʹ untranslated regions (*n*=36), in the coding sequence (*n*=5), within introns (*n*=51), and at the exon–intron boundary (*n*=3). The remaining 54 pre-miRNAs were annotated within the coding sequences of genes possessing annotated open reading frames; the majority of these were shorter than 150 amino acids in length and had no similarity to known proteins or protein motifs.

To learn more about the 107 pre-miRNAs that could have been encoded by independent transcriptional units, we compared transcriptomic data from MarpolBase covering each pre-miRNA from the 107 miRNAs and analyzed all available transcriptomic read coverage along the DNA sequences in which the pre-miRNAs were embedded. We combined RNA-seq and cap analysis gene expression sequencing (CAGE-seq) data to develop criteria for determining the transcription start site; the 3ʹend of the gene was defined as the last RNA-seq read aligned to the genomic sequence (Montgomery *et al.*, 2020). Our approach enabled us to identify gene structures for 24 pre-miRNAs representing 23 independent transcriptional units (Table 1); these comprised 15 intronless *MIR* genes, one polycistronic locus comprising two pre-miRNAs (Mpo-pre-miR390 and Mpo-pre-miR11676), and seven *MIR* genes containing a single intron. In the intron-containing genes, the miRNA stem-loop structure is predominantly located in the first exon, with a few notable exceptions. These include one gene (Mpo-*MIR11685*) in which the pre-miRNA is located within the second exon, and two genes (Mpo-*MIR11773* and Mpo-*MIR11815*) in which the precursor hairpin sequence is located within the intron. With regard to Mpo-*MIR11678*, two alternative pri-miRNA structures, resulting from alternative splicing, were identified. The longer Mpo-pri-miR11678.1 is over 2500 nt in length and was proposed based on RNA-seq reads profiled from antheridiophores; it is characterized by the presence of a 1108 nt long intron. The second pri-miRNA, Mpo-primiR11678.2, is shorter, containing a 125 nt long intron; this structure was identified based on the RNA-seq expression profiles of archegoniophores and heat-stress-treated plants. However, in both cases, the location of the pre-miRNA was the same, with both being located within the first exon. Since the level of Mpo-miR11678 is similar in both antheridiophores and archegoniophores, it is rather unlikely that the observed alternative splicing events affect the maturation efficiency of this miRNA (Tsuzuki *et al.*, 2016).

**Table 1.** Length and structure of 23 characterized *Marchantia polymorpha MIR* genes representing independent transcriptional units

Intronless Mpo-*MIR* genes						
*MIRNA* ID // gene ID		**Chromosome coordinates** ^**a**^ **(strand)**	**Gene length** ^***b***^	**Number of introns (length)**	**pre-miRNA coordinates**	**Location of miRNA**
Mpo-*MIR*11669 // Mp1g00675		chr1:739922..738280 (–)	1642 bp	–	739050..739217	–
Mpo-*MIR*11731 // Mp1g16315		chr1:16512588..16513713 (+)	1125 bp	–	16512919..16513019	–
Mpo-*MIR*11792 // Mp1g22755		chr1:23395553..23396596 (+)	1043 bp	–	23395668..23395757	–
Mpo-*MIR*11672 // Mp3g08375		chr3:8070378..8072664 (+)	2286 bp	–	8071432..8072344	–
Mpo-*MIR*11679 // Mp3g10085		chr3:9801184..9802171 (+)	987 bp	–	9801168..9801276	–
Mpo-*MIR*11687 // Mp3g17235		chr3:17303848..17305858 (+)	2011 bp	–	19805484..19805646	–
Mpo-*MIR*11777 // Mp5g04437		chr5:4930550..4928701 (–)	1850 bp	–	4930130..4930221	–
Mpo-*MIR*11865 // Mp5g15385		chr5:16515490..16517551 (+)	2062 bp	–	16516623..16516737	–
Mpo-*MIR*11700 // Mp5g24185		chr5:26156348..26154642 (–)	1707 bp	–	26155312..26155471	–
Mpo-*MIR*11695 // Mp6g03855		chr6:4268242..4266446 (–)	1797 bp	–	4267725..4267806	–
Mpo-*MIR*11680b // Mp6g07255		chr6:7960186..7959078 (–)	1109 bp	–	7959668..7959787	–
Mpo-*MIR*11680a // Mp6g18255		chr6:20474233..20475757 (+)	1524 bp	–	20474717..20474828	–
Mpo-*MIR*11674 // Mp7g15015		chr7:16671171..16669830 (–)	3380 bp	–	16670651..16670733	–
Mpo-*MIR*11717 // Mp7g16145		chr7:17775310..17773003 (–)	2308 bp	–	17774607..17774674	–
Mpo-*MIR*171 // Mp8g17875		chr8:20036988..20037680 (+)	693 bp	–	20037282..20037406	–
**Polycistronic Mpo-*MIR* genes**						
Mpo-*MIR*390 // Mp7g05245 Mpo-*MIR*11676 // Mp7g05247		chr7:6044481..6047405 (+)	2925 bp	–	6044934..6045065 6045158..6045246	–
**Intron-containing Mpo-*MIR* genes**						
***MIRNA* ID // Gene ID**		**Chromosome coordinates** ^**a**^ **(strand)**	**Gene length** ^***b***^	**Number of introns (length)**	**pre-miRNA coordinates**	**Location of miRNA**
Mpo-*MIR*11736 // Mp1g09755		chr1:10051644..10051081 (–) 10050900..10050043	1602 bp	1 (180 bp)	10051152..10051390	Exon 1
Mpo-*MIR*11678 ^***c***^	// Mp3g01145.1	chr3:1361154..1361555 (+) 1362664..1363718	2565 bp	1 (1108 bp)	1361250..1361517	Exon 1
	// Mp3g01145.2	chr3:1361154..1361555 (+) 1361681..1362220	1067 bp	1 (125 bp)	1361250..1361517	Exon 1
Mpo-*MIR*11732 // Mp3g24795		chr3:26326600..26324986 (–) 26324700..26323630	2971 bp	1 (285 bp)	26325832..26325925	Exon 1
Mpo-*MIR*11748a // Mp4g09365		chr4:10492516..10493163 (+) 10493315..10494272	1757 bp	1 (151 bp)	10492929..10493050	Exon 1
Mpo-*MIR*11773 // Mp5g16875		chr5:17855821..17855632 (–) 17855168..17854293	1529 bp	1 (463 bp)	17855250..17855342	Intronic
Mpo-*MIR*11815 // Mp6g01645		chr6:2176984..2176614 (–) 2171174..2169404	7581 bp	1 (5439 bp)	2173585..2173809	Intronic
Mpo-*MIR*11685 // Mp8g02425		chr8:2556749..2556447 (–) 2554181..2552850	3900 bp	1 (2265 bp)	2553976..2554099	Exon 2

All introns are of U2 type. The source of the transcription start site (^*a*^) are the combined RNA-seq and CAGE-seq data deposited in MarpolBase. The source of the longest 3ʹ end sequence (^*b*^) is the longest read from RNA-seq data deposited in MarpolBase. ^*c*^ Two alternative pri-miRNA structures were proposed based on specific RNA-seq reads profile deposited in MarpolBase.

All introns detected in the proposed gene structures were of the classical U2 type. However, for more than 80 of the 107 analyzed pre-miRNAs we were not able to propose a gene structure. There are two reasons for this. First, in many cases there was insufficient RNA-seq coverage in the genomic region where the analyzed pre-miRNA was located. Second, in some cases the miRNA precursor was found on the opposite strand from the corresponding annotated *M. polymorpha* gene. Future studies are therefore needed to clarify the specific structures of many *M. polymorpha MIR* genes.

When data published by Bowman *et al.* (2017) from 265 *M. polymorpha MIR* genes are taken into consideration, our analysis reveals that the majority of *M. polymorpha* miRNAs are encoded within protein-coding genes; this situation contrasts with that in *A. thaliana*, where the majority of *MIR* genes are independent transcriptional units (Szarzynska *et al.*, 2009; Bielewicz *et al.*, 2012; Zielezinski *et al.*, 2015). Unlike *M. polymorpha*, in *P. endiviifolia*, the characterized *MIR* genes represent independent transcriptional units. However, it should be taken into consideration that only 10 *MIR* gene structures are known for *P. endiviifolia* (Alaba *et al.*, 2015). This is also in contrast to *P. patens*, where, like in *M. polymorpha*, the majority of miRNA hairpins overlap with positions of annotated protein-coding loci (Axtell *et al.*, 2007). Moreover, our analysis identified one polycistronic locus in *M. polymorpha*, while in *P. patens* 25% of the miRNAs are polycistronic (Axtell *et al.*, 2007). Taking these data together, the organization of *MIR* genes in different bryophyte species differs considerably and may mirror the existence of additional regulatory layers that shape final miRNA levels.

## Higher plant miRNA biogenesis proteins are encoded within the *M. polymorpha* genome

For many years, researchers have focused investigations of genes and proteins from the microprocessor complex on angiosperms (Han *et al.*, 2004; Kurihara and Watanabe, 2004; Park *et al.*, 2005; Xie *et al.*, 2005; Lobbes *et al.*, 2006; Kurihara *et al.*, 2006). By contrast, much less is known about the specific structure and composition of such genes in early terrestrial plants. Genes encoding proteins found in the higher plant microprocessor core have previously been described in bryophytes. First, studies on the moss *P. patens* revealed the presence of all microprocessor core encoding genes, including *PpDCL1*, *PpSE*, and *PpHYL1.* Moreover, other important players in miRNA biogenesis, such as *PpHEN1* and *PpAGO1*, were also found (Arif *et al.*, 2013). The presence of several genes involved in miRNA biogenesis, including *DCL1*, *SE*, *HEN1*, and *AGO1*, was also reported in a representative of the most basal land plant lineage, the hornwort *Folioceros fuciformis* (You *et al.*, 2017). The existence of a *DCL1*-encoding gene was also discovered in the recently published genome of another hornwort, *A. angustus* (Li *et al.*, 2020; J. Zhang *et al.*, 2020). Our search for microprocessor complex-encoding genes in liverworts belonging to the three classes Haplomitriopsida, Jungermanniopsida, and Marchantiopsida, performed using the 1000 Plants platform (One Thousand Plant Transcriptomes Initiative, 2019), identified transcripts of key microprocessor proteins in the vast majority of liverwort species. These included DCL1, HYL1, and SE, as well as transcripts of genes encoding early miRNA biogenesis proteins such as Tough (TGH), Dawdle (DDL), and HEN1 (Table S2 at Zenodo). Since a large number of proteins involved in plant miRNA biogenesis have been identified thus far, we decided to assign these proteins into four sections. The first section focuses on the basic core proteins of the microprocessor (DCL1, SE, and HYL1), the second on auxiliary proteins that directly interact with microprocessor core components, the third focuses on selected regulatory proteins that indirectly affect the efficiency of miRNA biogenesis, and the fourth section describes proteins involved in miRISC formation. To understand more about the main liverwort miRNA biogenesis proteins, as well as the auxiliary and regulatory proteins that fine-tune miRNA production in higher plants, we searched the *M. polymorpha* genome (the only sequenced and annotated representative liverwort genome available) for orthologs of *A. thaliana* proteins that have been identified as being related to miRNA biogenesis.

### The core proteins of the microprocessor in *M. polymorpha*

In the *M. polymorpha* genome, several genes encoding core microprocessor proteins involved in miRNA biogenesis have previously been identified. These include Mp*DCL1a*, Mp*DCL1b*, and Mp*HYL1* (Lin *et al.*, 2016; Tsuzuki *et al.*, 2016; Bowman *et al.*, 2017; Lin and Bowman, 2018). The *SE* gene, which encodes one of the key components of the higher plant microprocessor, has thus far not been identified in the *M. polymorpha* genome. Our investigations revealed the existence of all genes that encode components of the plant core microprocessor, including Mp*SE*. Table 2 presents all identified *M. polymorpha* core protein genes as well as other genes that encode proteins interacting with the core microprocessor proteins.

**Table 2.** Proteins in *Marchantia polymorpha* orthologous to *Arabidopsis thaliana* proteins involved in miRNA biogenesis

***Arabidopsis thaliana***				Marchantia polymorpha			
**Protein name**	**Accession**	**Protein domains**	**Protein length (aa)**	**Accession**	**Protein domains**	**Protein length (aa)**	**Sequence identity (%)**
**Microprocessor core proteins (*n*=3)**							
DCL1	AT1G01040	ResIII—Helicase_C—Dicer_dimer—PAZ—Ribonuclease_3—Ribonuclease_3—DND1_DSRM	1910	Mp7g12090	ResIII—Helicase_C—Dicer_dimer—PAZ—Ribonuclease_3—Ribonuclease_3—DND1_DSRM	2057	61.5
		ResIII—Helicase_C—Dicer_dimer—PAZ—Ribonuclease_3—Ribonuclease_3—**DND1_DSRM**		Mp1g02840	ResIII—Helicase_C—Dicer_dimer—PAZ—Ribonuclease_3—Ribonuclease_3	1748	32.0
HYL1	AT1G09700	dsrm—dsrm	419	Mp7g08450	dsrm—dsrm	353	27.7
SE	AT2G27100	SERRATE_Ars2_N—ARS2	720	Mp1g23090	SERRATE_Ars2_N—ARS2	850	46.9
**Microprocessor auxiliary proteins (*n*=44)**							
APC10	AT2G18290	ANAPC10	192	Mp5g21650	ANAPC10	179	62.2
AtELP4	AT3G11220	PAXNEB	355	Mp3g07590	PAXNEB	387	42.8
CARP9	AT3G21290	Occludin_ELL	1192	Mp2g06020	Occludin_ELL	1627	24.9
CBP20	AT5G44200	RRM_1	257	Mp1g20560	RRM_1	238	67.6
CBP80	AT2G13540	MIF4G—MIF4G_like—MIF4G_like_2	848	Mp1g05560	MIF4G—MIF4G_like—MIF4G_like_2	876	51.4
CDC5	AT1G09770	Myb_DNA-bind_6—Myb_Cef	844	Mp1g10310	Myb_DNA-bind_6—Myb_Cef	954	56.5
CDF2	AT5G39660	zf-Dof	457	–	–	–	–
CHR2/BRM	AT2G46020	SNF2-rel_dom—Helicase_C	2193	–	–	–	–
CPL1	AT4G21670	NIF—dsrm—dsrm	967	Mp4g06900	NIF—dsrm—dsrm	986	42.5
DCL4	AT5G20320	**DEAD**—Helicase_C—Dicer_dimer—PAZ—Ribonuclease_3—Ribonuclease_3—DND1_DSRM	1702	Mp7g11720	**ResIII**—Helicase_C—Dicer_dimer—PAZ—Ribonuclease_3—Ribonuclease_3—DND1_DSRM	1932	29.6
DDL	AT3G20550	FHA	314	Mp7g08770	FHA	376	48.8
ELP2	AT1G49540	WD40—WD40—WD40—WD40—WD40—**WD40**	840	Mp8g01670	WD40—WD40—WD40—WD40—WD40	904	46.0
ELP5	AT2G18410	Elong_Iki1	374	Mp7g09690	Elong_Iki1	373	37.8
EMB2765	AT2G38770	Aquarius_N—AAA_11—AAA_12	1509	Mp6g16570	Aquarius_N—AAA_11—AAA_12	1648	60.4
FBW2	AT4G08980	F-box-like—LRR_6—LRR_6	317	Mp4g03420	F-box-like—LRR_6—LRR_6	310	32.2
HEN1	AT4G20910	Hen1_Lam_C—dsRBD2—**Methyltransf_31**	942	Mp3g16010	Hen1_Lam_C—dsRBD2—**Methyltransf_12**	1018	32.5
HEN2	AT2G06990	DEAD—Helicase_C—rRNA_proc-arch—DSHCT	995	Mp4g03900	DEAD—Helicase_C—rRNA_proc-arch—DSHCT	1009	68.6
HOS5	AT5G53060	KH_1—KH_1—KH_1	652	Mp3g0488	KH_1—KH_1—KH_1—**KH_1—KH_1**	721	36.4
ILP1	AT5G08550	GCFC	908	Mp8g18770	GCFC	995	40.9
KETCH1	AT5G19820	Importin_rep_4—HEAT_2—Importin_rep_6—HEAT	1116	Mp3g07820	Importin_rep_4—HEAT_2—Importin_rep_6—HEAT	1120	69
NOT2a	AT1G07705	NOT2_3_5	614	Mp1g21150	NOT2_3_5	723	48
NOT2b	AT5G59710	NOT2_3_5	614	–	–	–	–
NTR1	AT1G17070	TIP_N—G-patch—GCFC	849	Mp3g24100	TIP_N—G-patch—GCFC	848	49.6
				Mp3g09910	TIP_N—G-patch—GCFC	850	48.8
PPX1	AT4G26720	Metallophos	305	Mp2g13820	Metallophos	304	85.6
PRL1	AT4G15900	WD40—WD40—WD40—WD40—**WD40**	486	Mp6g09620	WD40—WD40—WD40—WD40	487	64.9
RACK1A	AT1G18080	WD40—WD40—WD40—WD40—WD40—WD40—WD40	327	–	–	–	–
RACK1B	AT1G48630	WD40—WD40—WD40—WD40—WD40—WD40—WD40	326	–	–	–	–
RACK1C	AT1G18080	WD40—WD40—WD40—WD40—WD40—WD40—WD40	326	Mp3g15630	WD40—WD40—WD40—WD40—WD40—WD40—WD40	316	71.2
RBM7	AT4G10110	RRM_1	173	Mp1g13100	RRM_1	220	30.0
RH6	AT2G45810	DEAD—Helicase_C	528	–	–	–	–
RH8	AT4G00660	DEAD—Helicase_C	505	Mp7g14570	DEAD—Helicase_C	515	74.7
RH12	AT3G61240	DEAD—Helicase_C	498	–	–	–	–
RH27	AT5G65900	DEAD—Helicase_C—DUF4217	633	–	–	–	–
RH42	AT1G20920	DEAD—Helicase_C	1166	Mp1g06750	DEAD—Helicase_C	1242	55.8
RS40	AT4G25500	RRM_1—RRM_1	350	–	–	–	–
RS41	AT5G52040	RRM_1—RRM_1	357	–	–	–	–
SAC3A	AT2G39340	SAC3_GANP	1006	Mp8g06380	SAC3_GANP	1142	37.9
SEAP1	AT4G24270	RRM_1—Lsm_interact	817	Mp8g05890	RRM_1—Lsm_interact	862	43.2
SIC	AT4G24500	–	319	Mp5g17530	–	278	23.8
SNRK2.6	AT4G33950	Pkinase	362	Mp1g24460	Pkinase	349	76.9
TGH	AT5G23080	DUF1604—Surp	930	Mp1g06200	DUF1604—Surp	1046	37.2
THO2	AT1G24706	THOC2_N—THOC2_N—Thoc2—Tho2	1823	Mp1g20320	THOC2_N—THOC2_N—Thoc2—Tho2	1978	52.7
THP1	AT2G19560	PCI	413	Mp1g04560	PCI	410	65.4
XCT	AT2G21150	XAP5	337	Mp2g07350	XAP5	333	79.2
ZCCHC8A	AT5G38600	PSP	532	Mp4g19730	PSP	675	25.2
**Microprocessor regulatory proteins (*n*=19)**							
COP1	AT2G32950	zf-C3HC4_2—WD40—WD40	675	Mp5g12010	zf-C3HC4_2—WD40—WD40	688	62.1
CPL2	AT5G01270	NIF—dsrm	774	–	–	–	–
EXPORTIN1A/XPO1A	AT5G17020	IBN_N—Xpo1—CRM1_repeat—CRM1_repeat_2—CRM1_repeat_3—CRM1_C	1075	Mp7g03970	IBN_N—Xpo1—CRM1_repeat—CRM1_repeat_2—CRM1_repeat_3—CRM1_C	1075	79.1
CRM1B/XPO1B	AT3G03110	IBN_N—Xpo1—CRM1_repeat—CRM1_repeat_2—CRM1_repeat_3—CRM1_C	1076				
FIP37	AT3G54170	Wtap	330	Mp1g24270	Wtap	364	44.2
GRP7	AT2G21660	RRM_1	176	Mp1g18780	RRM_1	193	62.1
HAKAI	AT5G01160	–	360	Mp7g09040	–	566	25
HESO1	AT2G39740	TUTase	511	–	–	–	–
HOS1	AT2G39810	ELYS	927	Mp7g02710	ELYS	780	35.4
HST1	AT3G05040	Xpo1—Exportin-5	1203	Mp2g09050	Xpo1—Exportin-5	1216	41.6
MOS2	AT1G33520	G-patch_2	462	Mp5g05110	G-patch_2	690	25.6
MPK3	AT3G45640	Pkinase	370	–	–	–	–
MTA	AT4G10760	MT-A70	685	Mp1g08870	MT-A70	816	50.2
MTB	AT4G09980	MT-A70	963	Mp1g04450	MT-A70	1520	29.9
PIF4	AT2G43010	HLH	472	–	–	–	–
PPX2	AT5G55260	Metallophos	346	–	–	–	–
SMA1	AT2G33730	DEAD—Helicase_C	733	Mp1g21580	DEAD—Helicase_C	935	54.9
STA1	AT4G03430	PRP1_N—TPR_14	1029	Mp6g10790	PRP1_N—TPR_14	942	69.1
VIR	AT3G05680	VIR_N	2138	Mp1g10290	VIR_N	2455	29.4
**miRISC formation proteins (*n*=3)**							
AGO1	AT1G48410	**Gly-rich_Ago1**—ArgoN—ArgoL1—PAZ—ArgoL2—ArgoMid—Piwi	1050	Mp1g18110	ArgoN—ArgoL1—PAZ—ArgoL2—ArgoMid—Piwi	1109	67.3
AGO4	AT2G27040	ArgoN—ArgoL1—PAZ—ArgoL2—**ArgoMid**—Piwi	924	Mp1g23190	ArgoN—ArgoL1—PAZ—ArgoL2—Piwi	943	40.4
AGO10	AT5G43810	ArgoN—ArgoL1—PAZ—ArgoL2—ArgoMid—Piwi	988	–	–	–	–

Proteins are classified into four sections (core, auxiliary, regulatory microprocessor, and miRISC formation proteins) and listed alphabetically in each section. Differences in domain architecture between Arabidopsis and *Marchantia polymorpha* orthologs are indicated in bold. Ortholog assignments between *A. thaliana* and *M. polymorpha* proteins were predicted by Orthofinder v. 2.5.4 using BLAST as the main sequence similarity search tool (Emms and Kelly, 2019). Pairwise alignments between orthologous protein sequences were calculated using the needle tool from the EMBOSS package (Rice *et al.*, 2000). Protein domains were annotated using the PfamScan tool and the Pfam 34.0 database (Mistry *et al.*, 2021).

### Selected microprocessor auxiliary proteins in *M. polymorpha*

Next, we searched the *M. polymorpha* genome for genes coding for auxiliary proteins that are known to affect miRNA biogenesis in higher plants by interacting with core microprocessor components (Table 2). Here, we identified Mp*TGH*, which encodes an ortholog of Arabidopsis RNA-binding TGH protein. In *A. thaliana*, this protein is known to be involved in the early stages of miRNA biogenesis and to interact with DCL1 and mRNA adenosine methylase (MTA), which introduces m6A into mRNA and pri-miRNAs (Table 2). TGH is also known to bind to both pri-miRNAs and pre-miRNAs and is necessary for the effective interaction of pri-miRNA with HYL1 (Ren *et al.*, 2012; Bhat *et al.*, 2020). We also identified Mp*DDL*, which encodes a conserved forkhead-associated (FHA) domain-containing protein that in Arabidopsis interacts directly with the DCL1 protein, thereby affecting pri-miRNA processing (S. Zhang *et al.*, 2018).

In *A. thaliana*, it has been shown that the Cell Division Cycle 5-like (CDC5), Protein Pleiotropic Regulatory Locus 1 (PRL1), and RNA helicase MAC7 proteins form a MOS4-associated complex (MAC) that interacts with DCL1/SE, pri-miRNAs, and HYL1 (Zhang *et al.*, 2013; Jia *et al.*, 2017; Manavella *et al.*, 2019). Here, CDC5 and PRL1 interactions modulate pri-miRNA levels, resulting in miRNA accumulation, and work in concert as a complex to increase DCL1 activity (Zhang *et al.*, 2013, 2014). We identified genes encoding all MAC components in *M. polymorpha*, including Mp*CDC5*, Mp*PRL1*, and Mp*MAC7*.

We also found an ortholog of one of the intrinsically disordered proteins that in *A. thaliana* has been designated Constitutive Alterations in the small RNAs Pathways 9 (CARP9). We designated the gene identified in the *M. polymorpha* genome as Mp*CARP9*. In Arabidopsis, the CARP9 protein is known as a novel miRNA pathway partner. It interacts with HYL1 and AGO1, leading to efficient miRNA loading on AGO1 (Tomassi *et al.*, 2020).

The presence of a *M. polymorpha* ortholog of the Negative On TATA Less 2 (NOT2) protein is another example of a microprocessor-interacting protein that is shared by *A. thaliana* and *M. polymorpha*. NOT2 operates as a general factor during miRNA biogenesis in angiosperms, where it promotes miRNA gene transcription and facilitates effective DCL1 recruitment through their direct interaction (Wang *et al.*, 2013). Unlike *A. thaliana*, which possesses two paralog proteins, NOT2a (AT1G07705) and NOT2b (AT5G59710), in *M. polymorpha* only one gene encoding NOT2a (Mp*NOT2a*) is present (Table 2).

In addition, the Nuclear cap-binding protein subunit 1 (CBP80) and Nuclear cap-binding protein subunit 2 (CBP20) proteins found in *A. thaliana* form a nuclear cap-binding complex (CBC) and are involved in pri-miRNA biogenesis and splicing control by directly interacting with the SE protein (Laubinger *et al.*, 2008; Kim *et al.*, 2008). In our analysis of the *M. polymorpha* genome, we identified genes that encode both CBC subunits (Mp*CBP20* and Mp*CBP80*).

Speth *et al.* (2013) reported that the Receptor of Activated Kinase (RACK1) may interact with SE, suggesting that RACK1 plays a role in *A. thaliana* miRNA processing. The RACK1 protein is known to contain beta-transducin (WD40) repeats and therefore is highly conserved among plant species (Ullah *et al.*, 2008; Speth and Laubinger, 2014). In Arabidopsis, this gene has three paralogs known as *RACK1A*, *RACK1B*, and *RACK1C*. However, in the *M. polymorpha* genome we were able to identify only Mp*RACK1C*.

The *A. thaliana* HEN1 protein was identified as an RNA methyltransferase that methylates the 3ʹ ends of the miRNA/miRNA* duplex. This protein is also known to interact directly with DCL1 and HYL1 (Li *et al.*, 2005; Yu *et al.*, 2005; Baranauske *et al.*, 2015). We identified an Mp*HEN1* protein-coding gene in the *M. polymorpha* genome (Lin *et al.*, 2016; Tsuzuki *et al.*, 2016; Bowman *et al.*, 2017).

Finally, in *M. polymoprha* we identified *Serrate Associated Protein 1* (Mp*SEAP1*) gene which encodes an ortholog of *A. thaliana* SEAP1 protein. In Arabidopsis SEAP1 interacts with SE and affects the splicing and early biogenesis stages of a set of miRNAs (M. Li *et al.*, 2021).

We were unable to identify orthologs of some protein-coding genes that are known to affect miRNA biogenesis in Arabidopsis by directly interacting with microprocessor core elements. Of these, we did not find *Chromatin remodelling factor 2* (*CHR2*, also known as *Brahma*, *BRM*), which encodes one of the two catalytic ATPase subunits of the chromatin remodeling complex. The CHR2 protein remodels the secondary structure of miRNA primary transcripts in *A. thaliana* and consequently down-regulates miRNA production (Wang *et al.*, 2018; Thouly *et al.*, 2020). In *A. thaliana*, Cycling DOF factor 2 (CDF2) interacts with DCL1 and acts in the same pathway as miR156 or miR172 to control flowering (Sun *et al.*, 2015). The absence of CDF2 in *M. polymorpha* may be linked to the absence of miR156 and miR172.

### Selected microprocessor regulatory proteins in *M. polymorpha*

Here we report the identification of selected protein-coding genes known to affect miRNA biogenesis in higher plants that indirectly interact with microprocessor core components.

It was recently found that the HST1 protein is involved in the early stages of miRNA biogenesis and participates in the cell-to-cell transfer of mature miRNAs in *A. thaliana* (Brioudes *et al.*, 2021; Cambiagno *et al.*, 2021). An orthologous *HST1* gene is present in the *M. polymorpha* genome.

mRNA adenosine methylase (MTA) was identified as a general methyltransferase that introduces m6A to RNA Pol II transcripts in Arabidopsis (e.g. mRNA and pri-miRNA) (Zhong *et al.*, 2008; Bhat *et al.*, 2020). MTA deficiency results in the down-regulation of a set of miRNAs by causing inefficient recruitment of microprocessor components related to chromatin; this therefore affects pri-miRNA structure (Bhat *et al.*, 2020). We identified a gene encoding MTA in the *M. polymorpha* genome (Mp*MTA*). Moreover, we also identified all components of the m6A methyltransferase complex in *M. polymorpha* [MTA, N6-adenosine-methyltransferase non-catalytic subunit MTB (MTB), FKBP12-interacting protein of 37 kDa (FIP 37), Protein virilizer homolog (VIR), and E3 ubiquitin-protein ligase Hakai (HAKAI)].

Recently, it has been shown that different DEAD-box helicases play important roles as splicing factors to control miRNA biogenesis (Li *et al.*, 2018; Li and Yu, 2021; Hou *et al.*, 2021). For instance, genes encoding Small 1 (SMA1) and RNA helicase 8 (RH8) have been identified as Mp*SMA1* and Mp*RH8* within the *M. polymorpha* genome. According to Q. Li *et al.* (2021), Arabidopsis RH8 (together with RH6 and RH12) controls the formation of dicing-bodies (D-bodies) by interacting with the SE protein (Li *et al.*, 2018). However, we did not identify RH6 and RH12 in the *M. polymorpha* genome. It would therefore be interesting to study whether the MpRH8 protein is implicated in the formation of D-bodies in *M. polymorpha*.

Yan *et al.* (2017) reported that SNF1-Related Protein Kinase subfamily 2 (SnRK2) proteins are required for the proper accumulation of miRNAs in *A. thaliana* by regulating miRNA processing factors. Specifically, the authors showed that the function of the HYL1 protein in Arabidopsis depends on SnRK2 kinases when plants experience osmotic stress. The *M. polymorpha* genome contains two genes encoding SnRK2 proteins, Mp*SnRK2A* and Mp*SnRK2B* (Bowman *et al.*, 2017). However, their putative role in miRNA biogenesis in *M. polymorpha* requires experimental validation.

### Selected proteins forming the miRISC complex

The AGO1 protein as a component of the miRISC complex, guided by miRNA, cleaves target mRNAs at a specific site, namely between the 10th and 11th nucleotides of the miRNA sequence (counting from the 5ʹ end). It has previously been reported that the *M. polymorpha* genome encodes an MpAGO1 protein (Lin 2016; Tsuzuki *et al.*, 2016; Lin and Bowman, 2018). Another important protein from the Argonaute family known from Arabidopsis studies is AGO10. This protein acts as a negative regulator of *AGO1* transcript levels. Moreover, it specifically binds miR165/166 targeting HD-ZIP III transcription factor mRNAs. Its presence is known to be critical for proper shoot apical meristem formation (Zhu *et al.*, 2011; Yu *et al.*, 2017). The previously reported absence of AGO10 in *M. polymorpha* is especially intriguing due to the presence of miRNA165/166 in its genome (Lin *et al.*, 2016; Tsuzuki *et al.*, 2016).

In conclusion, the set of conserved miRNAs in terrestrial plants is limited in number. Moreover, while individual miRNAs appear to be highly species specific, the proteins involved in the microprocessor, as well as those proteins that interact with it directly or indirectly, are very similar and likely originated very early during the evolution of land plants.

## *M. polymorpha* orthologs of *A. thaliana* proteins involved in miRNA biogenesis exhibit the same domain architecture

We investigated the orthologous genes in *M. polymorpha* and Arabidopsis that encode proteins involved in miRNA biogenesis. Specifically, we looked for *M. polymorpha* sequences that are orthologous to the Arabidopsis microprocessor core proteins (*n*=3; DCL1, HYL1, and SE), auxiliary (*n*=45), and regulatory (*n*=19) proteins, as well as miRISC formation proteins (*n*=3; AGO1, AGO4, and AGO10). In total, out of 70 Arabidopsis proteins involved in miRNA biogenesis, we found 54 orthologs (77%) in *M. polymorpha* (Table 2).

**Fig. 4.**
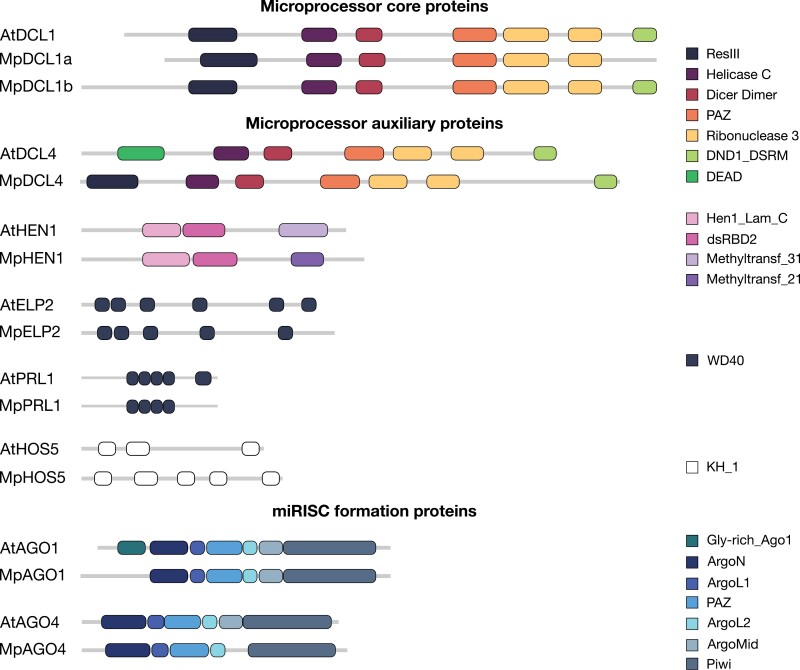
Differences in domain architecture between Arabidopsis and *M. polymorpha* orthologs involved in miRNA biogenesis. Protein domains were annotated using the PfamScan tool and the Pfam database (Mistry *et al.*, 2021). The figure includes the following Pfam accession numbers: ArgoL1 (PF08699), ArgoL2 (PF16488), ArgoMid (PF16487), ArgoN (PF16486), DEAD (PF00270), Dicer_dimerm (PF03368), DND1_DSRM (PF14709), dsRBD2 (PF17842), Gly-rich_Ago1 (PF12764), Helicase C (PF00271), Hen1_Lam_C (PF18441), KH_1 (PF00013), Methyltransf_21 (PF05050), Methyltransf_31 (PF13847), PAZ (PF02170), Piwi (PF02171), RESIII (PF04851), Ribonuclease 3 (PF00636), and WD40 (PF00400).

The three core microprocessor proteins (DCL1, HYL1, and SE) in *M. polymorpha* have the same sequential arrangement of domains along their protein sequence (i.e. domain architecture) as the corresponding Arabidopsis proteins. The genome of *M. polymorpha* was previously found to encode two DCL1 protein genes, Mp*DCL1a* and Mp*DCL1b*, orthologous to DCL1 from Arabidopsis (Lin *et al.*, 2016; Tsuzuki *et al.*, 2016; Bowman *et al.*, 2017). However, our analysis revealed that *AtDCL1* shares an almost two-fold higher sequence identity with Mp*DCL1b* (62%) than with Mp*DCL1a* (32%). Moreover, MpDCL1b and AtDCL1 proteins share the same domain architecture, whereas MpDCL1a protein lacks the C-terminal domain that binds double-stranded RNA (DND1_DSRM) (Table 2, Fig. 4). Although two other microprocessor core proteins, HYL1 and SE, are less conserved than DCL1 in terms of sequence identity with their Arabidopsis orthologs (28% and 47%, respectively), both the MpHYL1 and MpSE proteins have a common domain architecture with the corresponding AtHYL1 and AtSE proteins, implying functional similarity of the orthologs (Table 2). Notably, the low sequence identity between AtHYL1 and MpHYL1 is due to high sequence variability at the C-terminus of both proteins: AtHYL1 contains six tandem repeats of 28 amino acid motifs, a pattern that is not observed in MpHYL1. However, it should be noted that repetitive extension of AtHYL1 is a characteristic feature of proteins found only in Brassicaceae (Lu and Fedoroff, 2000).

Most of the microprocessor auxiliary proteins reported in Arabidopsis (*n*=45) are also present in *M. polymorpha* (*n*=34; 77%). The protein sequences of the microprocessor auxiliary orthologs are similar in length and their mean sequence identity is 51% (±17% standard deviation). The highest sequence identity between orthologs is observed for Protein Phosphatase X (PPX) (86%), XAP5 Circadian Timekeeper (XCT) (79%), and SNRK2.6 (76%) proteins, and the least conserved sequences include Sickle (SIC) (24%), CARP9 (25%), and the scaffold zinc-knuckle protein (ZCCHC8A) (25%). Although AtSIC and MpSIC lack recognizable protein domains, both proteins were predicted as orthologs, suggesting their functional coherence. Most auxiliary microprocessor orthologs between Arabidopsis and *M. polymorpha* preserve the same domain content and order; however, five of them—DCL4, HEN1, High Osmotic Stress Gene Expression 5 (HOS5), Elongator complex protein 2 (ELP2), and PRL1—show differences in their domain architecture (Fig. 4, Table 2). These differences include either small variations in the number of domain copies (e.g. five copies of the WD40 domain in AtPRL1 and four copies in MpPRL1) or changes in domain content. The alterations in domain content relate to the absence of one domain in one of the orthologs. Specifically, in the DCL4 and HEN1 proteins, one domain in one ortholog seems to be replaced with another functionally similar domain in the corresponding sequence (DEAD with ResIII and Methyltransf_31 with Methyltransf_21, respectively). Given that alterations in domain architecture can impact protein function, we cannot exclude the possibility that the proteins in *M. polymorpha* with different domain compositions have acquired or lost some functionality (Hegyi and Gerstein, 2001). Functional studies are needed to verify this hypothesis. As such, a recent paper investigating the functional comparison of HEN1 proteins from Arabidopsis and *M. polymorpha* has shown that MpHEN1 protein activity is comparable with AtHEN1, and both proteins show substrate specificity toward duplex sRNA. Consistently, MpHEN1 structure modeling based on the AtHEN1 crystal structure revealed conservation of the *M. polymorpha* protein architecture, with several critical motifs responsible for RNA binding and methylation being highly conserved (Sanobar *et al.*, 2021).

Furthermore, most of the regulatory microprocessor proteins reported for Arabidopsis (*n*=19) are also present in *M. polymorpha* (*n*=14; 74%) (Table 2). The level of sequence conservation of these proteins (sequence identity: 47% ±17%) is very similar to that observed for auxiliary microprocessor orthologs. In addition, all microprocessor regulatory orthologs preserve the same domain architecture. The prime example is Chromosome region maintenance 1 (Exportin-1) MpCRM1 (MpXPO1), which shows a very high sequence identity of 79% with both AtExportin1A (AtXPO1A) and AtCRM1B (AtXPO1B) proteins and also preserves the same arrangement of six different domains.

In the case of miRISC formation proteins (AGO1, AGO4, and AGO10), we found orthologs of AGO1 and AGO4 between Arabidopsis and *M. polymorpha*. At the protein sequence level, the AGO1 orthologs show higher identity (67%) than the AGO4 orthologs (40%). We also observed differences at the protein domain level: MpAGO1 lacks a recognizable N-terminal glycine-rich domain that is present in AtAGO1, while MpAGO4 lacks a Mid domain, which is part of the Piwi lobe of AtAGO4.

Finally, we were unable to identify *M. polymorpha* orthologs for 16 Arabidopsis proteins, including 10 auxiliary microprocessor proteins [e.g. CHR1, CDF, two serine/arginine-rich splicing factors (RS40/41)], five regulatory microprocessor proteins [e.g. HEN1 Suppressor1 (HESO1), Mitogen-Activated Protein Kinase 3 (MPK3), PPX2], and AGO10 protein belonging to miRISC formation proteins (Table 2). We suggest that either these proteins are not encoded by the *M. polymorpha* genome or their corresponding amino acid sequences lack sufficient similarity for them to be assigned as orthologs. However, as has been reported (Bowman *et al.*, 2017), some of the Arabidopsis proteins represent multigene families that encompass more protein members than the corresponding proteins found in *M. polymorpha*. For example, the Arabidopsis genome encodes three RACK proteins, whereas *M. polymorpha* has only one RACK member. In addition, the *RH* gene family is more expanded in Arabidopsis (i.e. *RH6*, *RH8*, *RH12*, *RH27*, and *RH42*) than in *M. polymorpha* (*RH8* and *RH42*).

## Concluding remarks

Bryophytes are thought to have shared a common ancestor with higher plants, and miRNAs may have had important regulatory functions before their divergence. In this review, we have summarized the available data on *MIR* gene structures, miRNA biogenesis, and miRNA-mediated gene regulation analysis datasets. These demonstrate both significant similarities and important differences between Bryophyta and higher plants. Conserved miRNA families in *M. polymorpha* (and other Bryophyta species) are limited; most miRNA families appear to be highly species specific. In particular, we show that *M. polymorpha* and *P. endiviifolia* share only three miRNA families. Moreover, in contrast to the higher plants, in which *MIR* genes are usually found in intergenic regions, most identified pre-miRNAs in *M. polymorpha* overlap with protein-coding genes. By identifying gene structures representing independent transcriptional units, we found that *M. polymorpha MIR* genes are mostly intronless. As in angiosperms, the majority of liverwort-identified miRNAs appear to target transcription factors and therefore play a role in the corresponding regulatory pathway. With respect to miRNA biogenesis, the microprocessor core protein composition and its auxiliary and regulatory proteins in *M. polymorpha* are highly conserved. We also found a high degree of similarity between the domain architecture in *M. polymorpha* and in Arabidopsis. However, we also found important exceptions, where crucial players involved in miRNA biogenesis differ at selected domains (including the AGO1, HEN1, and DCL1a proteins). These observations require additional studies to determine the functional consequences of these differences. All data summarized in this review confirm the hypothesis that miRNA-mediated silencing machinery emerged during the early stages of plant evolution. Future studies on the microtranscriptomes of *M. polymorpha* and other Bryophyta are necessary to provide a better understanding of land plant evolution. Moreover, such studies may help answer questions about how land plants have adapted to the terrestrial environment.

## Data Availability

Data associated with the manuscript are openly available at Zenodo: https://doi.org/10.5281/zenodo.6327001; Pietrykowska *et al.* (2022).
